# A Targeted Liposomal Delivery System for Hydrophobic Antitumor Compounds Using the Example of a 1,2,3,4-Tetrahydroisoquinoline Derivative

**DOI:** 10.32607/actanaturae.28027

**Published:** 2026

**Authors:** G. M. Proshkina, O. N. Ashirov, M. N. Zharkov, A. Al-Khadj Aioub, V. I. Vinogradova, D. Zhu, R. Yan, Sh. S. Azimova, E. I. Shramova, N. A. Pyataev, S. M. Deyev

**Affiliations:** Shemyakin–Ovchinnikov Institute of Bioorganic Chemistry, Russian Academy of Sciences, Moscow, 117997 Russia; Yunusov Institute of the Chemistry of Plant Substances, Academy of Sciences of the Republic of Uzbekistan, Tashkent, 100170 Uzbekistan; Ogarev National Research Mordovia State University, Saransk, 430005 Russia; Center of Advanced Pharmaceuticals and Biomaterials, China Pharmaceutical University, Nanjing, 211198 China; School of Science, China Pharmaceutical University, Nanjing, 211198 China; Moscow Engineering Physics Institute, National Research Nuclear University “MEPhI”, Moscow, 115409 Russia

**Keywords:** liposomal delivery system, HER2-positive adenocarcinomas, tetrahydroisoquinolines

## Abstract

Cancer is the second leading cause of death worldwide, after cardiovascular disorders. One of the main constraints of anticancer agents is their limited water solubility, which complicates preclinical *in vivo* studies. In this work, we propose a versatile approach for stabilizing hydrophobic antitumor agents using liposomes based on natural phospholipids. The efficacy of the method is demonstrated for hydrophobic 1-tridecyl-6,7-dimethoxy-1,2,3,4-tetrahydroisoquinoline, a promising tetrahydroisoquinoline derivative with proven antitumor activity against lymphoid and epithelial cancer cells. We show that the liposomal formulation ensures drug stability in aqueous media. Modification of the liposome surface with a HER2-specific non-immunoglobulin module strongly improved targeting efficiency. The targeting index differed markedly between cells with low versus high HER2 expression levels (3.02 and 18.98, respectively), demonstrating that the targeted liposomal formulation exerts a substantially enhanced cytotoxicity against HER2-overexpressing cells. The proposed liposomal platform can also be utilized to deliver other hydrophobic anticancer drugs.

## INTRODUCTION

Despite the impressive recent advances achieved in cancer treatment, the disease continues to be one of the most pressing issues in contemporary medicine. Adverse effects, primary or acquired tumor resistance, and lack of treatment effectiveness are the key challenges associated with modern anticancer therapy.

1,2,3,4-Tetrahydroisoquinoline (THIQ) derivatives are compounds with proved anticancer activity that are the subject of extensive investigation in medicinal chemistry. When used as antitumor agents, THIQ derivatives can inhibit antiapoptotic proteins (BCL-2, BCL-XL, and MCL-1), as well as the enzymes that remodel the DNA and histone architecture, modulate the activity of estrogen receptors, and inhibit the ABC transporters (P-gp, BCRP, and MRP1) that are responsible for the development of multiple drug resistance in cancer cells [1, 2, 3, 4, 5].

However, the hydrophobic nature of these compounds significantly limits their *in vivo* applications, which prioritizes the development of novel drug formulations.

Enhancing the solubility of hydrophobic drugs is an important objective in modern pharmacology, since it is this very factor that is largely responsible for their bioavailability and effectiveness.

Since their discovery by Bangham [6], liposomes remain one of the most efficacious drug delivery vehicles [7] owing to their biocompatibility, lack of intrinsic toxicity, the ability to encapsulate large quantities of drugs both in the aqueous and hydrophobic phases, and the feasibility of modifying the outer surface of the liposomes with ligands specific to tumor-associated antigens [8, 9, 10, 11, 12].

Earlier, a panel of 1,2,3,4-tetrahydroisoquinoline derivatives was synthesized at the Yunusov Institute of the Chemistry of Plant Substances (Tashkent, Uzbekistan) in order to screen anticancer activity [13]. Among the synthesized compounds, 1-tridecyl-6,7-dimethoxy-1,2,3,4-tetrahydroisoquinoline was found to exhibit the highest *in vitro* anticancer activity against various cancer cells. However, further *in vivo* studies were impeded by the high hydrophobicity of this compound.

In this work, we propose using the method of incorporating 1-tridecyl-6,7-dimethoxy-1,2,3,4-tetrahydroisoquinoline into liposomes exhibiting tropism for the prognostically significant tumor-associated antigen HER2 (human epidermal growth factor receptor 2) to solve the solubility problem and ensure targeted delivery of the drug to tumor cells. DARPin_9-29 (designed ankyrin repeat protein), which is a non-immunoglobulin protein scaffold based on ankyrin repeat motifs and an antibody mimetic specifically targeting HER2 subdomain I, was used as a vector molecule guiding liposomes to a specific target [14]. The experimental design is shown in *Fig. 1*.

**Fig. 1:**
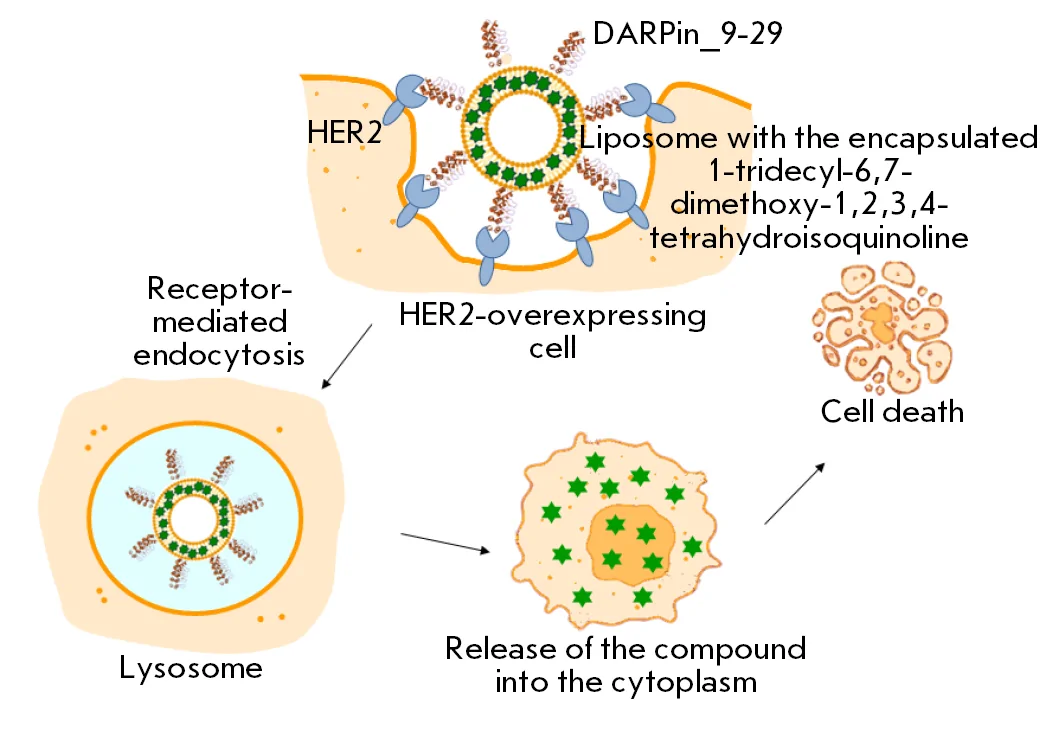
The concept of targeted liposomal delivery of the hydrophobic anticancer compound 1-tridecyl-6,7-dimethoxy-1,2,3,4-tetrahydroisoquinoline. DARPin-modified liposomes with 1-tridecyl-6,7-dimethoxy-1,2,3,4-tetrahydroisoquinoline incorporated into the lipid bilayer specifically interact with the extracellular domain of HER2 on the surface of cancer cells and are internalized via receptor-mediated endocytosis, followed by the release of the compound into the cytoplasm and induction of cell death

The findings obtained for *in vitro* cell cultures demonstrate that the DARPin-modified liposomal formulation of 1-tridecyl-6,7-dimethoxy-1,2,3,4-tetrahydroisoquinoline is stable in aqueous media, efficiently binds to HER2 on the surface of cancer cells, is internalized within 2 h, and is characterized by highly specific cytotoxicity against HER2-positive cells.

## EXPERIMENTAL

**Synthesis of DARPin-modified liposomes loaded with 1-tridecyl-6,7-dimethoxy-1,2,3,4-tetrahydroisoquinoline**

Liposomes loaded with 1-tridecyl-6,7-dimethoxy-1,2,3,4-tetrahydroisoquinoline (below referred to as compound #1, *Fig. 2*) were synthesized using the thin-film hydration method. Compound #1 (4 mg; 0.34 mg/mL) was added to a lipid mixture (270 mg of egg lecithin, 15 mg of cholesterol, and 30 mg of phosphatidylethanolamine, 85% : 5% : 10%); all the components were dissolved in 15 mL of chloroform.

**Fig. 2:**
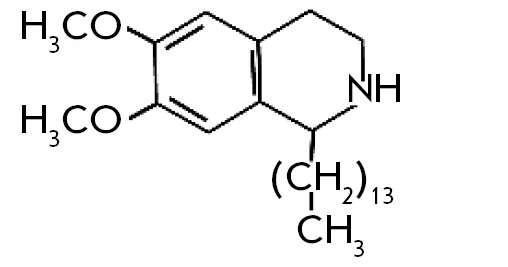
Structural formula of 1-tridecyl-6,7-dimethoxy-1,2,3,4-tetrahydroisoquinoline (compound #1)

The mixture was concentrated on a rotary evaporator at 40–45°C. In order to thoroughly remove the residual organic solvent, a flow of nitrogen was gently purged through the flask containing the film for 5 min. Lipid film hydration was conducted using 10 mL of a sterile 0.9% NaCl solution under constant stirring at 30°C. The hydration was continued until the films had completely detached from the flask walls and a milky white turbid suspension of multilamellar vesicles had formed.

Liposomes of a certain size were obtained by extruding the resulting suspension through polycarbonate membranes with pore sizes of 400, 200, and 100 nm. Extrusion was repeated several times (5 cycles) using an Avanti mini extruder. Dialysis against a 0.9% NaCl solution was performed for 3 days to remove compound #1 not encapsulated by liposomes.

The resulting liposomes were conjugated to the HER2-targeted module DARPin_9-29 using Traut’s reagent and the sulfo-EMCS hetero-bifunctional cross-linking agent, according to the procedure described in ref. [9].

The hydrodynamic diameter and ζ-potential of DARPin-modified and non-modified liposomes loaded with compound #1 were determined using a Zetasizer Nano ZS analyzer (Malvern Instruments). Prior to measurements, the samples in the solution containing 150 mM NaCl, 20 mM NaPi, pH 7.5, were diluted 25-fold with water.

**Measuring the 1-tridecyl-6,7-dimethoxy-1,2,3,4-tetrahydroisoquinoline content in the liposomes**

Dialysis against a 0.9% NaCl solution for 3 days at room temperature, with the dialysis fluid replaced daily, was performed in order to remove the non-encapsulated compound #1. The concentration of compound #1 in the liposomes was measured spectrophotometrically after liposome disruption by methanol relative to a reference (non-loaded liposome emulsion with the same mass concentration of lipids). The initial concentration of compound #1 upon liposome synthesis was 0.874 mM (0.34 g/L); the total lipid concentration was 34 mM (25.2 g/L). The encapsulation efficiency was assessed as the ratio between the concentration of compound #1 in liposomes after dialysis and their initial concentration. The encapsulation efficiency was ≥ 95%.

**Conjugation of DARP-Lip(#1) and DARPin_9-29 to the AF-488 dye**

DARP-Lip(#1) and DARPin_9-29 were conjugated to the AF-488 hydroxy succinimide ester (Lumiprobe, Russia), to be further used in flow cytometry and confocal microscopy. The conjugation of DARP-Lip(#1) and DARPin_9-29 to the dye was conducted at room temperature for 1 h in 20 mM NaPi, pH 8.0, and 150 mM NaCl in the presence of a 10-fold molar excess of the dye. The non-conjugated dye was separated from the protein by gel filtration on a NAP5 column (Cytiva, USA) equilibrated with a buffer containing 20 mM NaPi, pH 7.5, and 150 mM NaCl. DARP-Lip(#1) and DARPin_9-29, modified with AF-488, were collected in the column void volume.

**Cell cultures**

Human ovarian carcinoma (SKOV-3) and human cervical carcinoma (HeLa) cells, as well as the immortalized mouse fibroblast L929 cells, were used in this study. The cells were cultivated at 37°C in a moist atmosphere containing 5% CO_2_ in the RPMI medium (PanEco, Russia) supplemented with 2 mM L-glutamine (PanEco), 10% fetal bovine serum (Gibco, USA), and an antibiotic (10 U/mL penicillin, 10 µg/mL streptomycin, PanEco).

**Flow cytometry and confocal microscopy**

The DARP-Lip(#1)–HER2 interaction was assessed on the SKOV-3 and HeLa cell lines by flow cytometry or confocal microscopy.

For the flow cytometry experiments, the cells were incubated in the presence of AF-488-modified DARPin_9-29 or DARP-Lip(#1) (250 nM; the concentration was calculated on the basis of the dye) in a complete growth medium at 37°C for 10 min. After the incubation, the cells were washed thrice with PBS and analyzed using a NovoCyte 3000 flow cytometer. The fluorescence of AF-488 was excited using a 488-nm laser; fluorescence was detected in the 530 ± 30 nm channel (the FITC channel).

For the confocal microscopy experiments, the cells were seeded into the wells of a 96-well glass-bottom microplate (Cell imaging plates, 96 wells, Eppendorf) 24 h prior to the experiment at a density of 35,000 cells/mL (100 µL/well). The cells were then incubated in the presence of the 250 nM DARP-Lip(#1) conjugate (concentration calculated on the basis of the dye) for 20 or 120 min. The nuclei were stained with 10 nM Hoechst 33342 (10 min at 37°C). After washing the cells thrice with PBS and adding the FluoroBright medium (Gibco), the cells were analyzed using an LSM 980 confocal microscope (Zeiss) with a 63× Plan-Apochromat oil-immersion objective. The fluorescence of Hoechst 33342 was excited using a 405-nm laser and detected at 410–520 nm. The fluorescence of AF-488 was excited using a 488-nm laser and detected at 497–562 nm.

**Studying the stability of the free and liposomal formulations of compound #1 in aqueous media**

The aggregation of the free and liposomal formulations of compound #1 was studied in the mouse fibroblast L929 culture. The cells were seeded into the wells of a 96-well microplate (5 × 10^4^ cells/mL) and cultured overnight under standard conditions. After compound #1 (free and liposomal formulations) at concentrations of 400, 100, and 12.5 µM had been added, the cells were further cultivated for 24 h. Transmitted light microscopy was performed using a Nexcope NIB900 inverted light microscope coupled to an FL20 Tuscen digital camera.

***In vitro* cytotoxicity**

The cytotoxic effect of compound #1 on the SKOV-3 and HeLa cell lines was measured by the MTT assay [15]. Cells were seeded into 96-well plates (SKOV-3 – 3 × 10^4^ cells/mL; HeLa – 2.5 × 10^4^ cells/mL) and cultivated for 16 h under standard conditions. The growth medium was then replaced with a fresh one containing HER2-targeted or non-targeted liposomes at different concentrations, loaded with compound #1. Incubation in the presence of liposomes was conducted for 72 h.

Cell viability was measured using 0.5 mg/mL of a 3-(4,5-dimethylthiazol-2-yl)-2,5-diphenyltetrazolium bromide solution. The cells were incubated at 37°C for 1 h, the growth medium was removed, and formazan crystals were dissolved in 100 µL of dimethyl sulfoxide. Absorbance of the solution was measured at 570 nm using an Infinite M1000 microplate reader (Tecan, Switzerland) equipped with a monochromator.

The relative cell viability was calculated as the ratio between the averaged absorbance in the wells containing treated and non-treated (control) cells and expressed as a percentage. The concentration of the test compound required to reduce relative cell viability by 50% compared to the control (IC_50_) was calculated using the GraphPad Prism 10.1.0 software by nonlinear regression with a four-parameter dose–response model.

The targeting index was calculated as the ratio between the IC_50_ values of non-targeted liposomes and HER2-specific liposomes synthesized under identical conditions using the same cell lines.

## RESULTS AND DISCUSSION

**Synthesis and characterization of HER2-specific liposomes loaded with 1-tridecyl-6,7-dimethoxy-1,2,3,4-tetrahydroisoquinoline**

Due to their ability to encapsulate hydrophobic compounds at high concentrations, liposomes hold promise as a means for enhancing drug bioavailability. Furthermore, the feasibility of tailoring their composition is among the most valuable advantages of liposomes as drug delivery platforms.

Liposomes were produced using the main phospholipid components of biological membranes, with their fluidity modulated by the presence of cholesterol. Phosphatidylethanolamine contained in the lipid mixture allows one to modify the outer liposome surface using a HER2-specific targeted module.

Compound #1 was loaded into liposomes according to the procedure described in the Experimental section. The advantages of liposomal encapsulation of compound #1 are evident upon incubation with cells: the free compound #1 added to the growth medium containing L929 cells instantaneously precipitated yielding crystals clearly discernible by transmitted light microscopy. The tendency to form aggregates makes it impossible to properly assess cytotoxicity. Contrariwise, the liposome-encapsulated compound neither precipitated nor formed crystalline structures under the same conditions, thus indicating that the hydrophobic compound was stabilized within the lipid bilayer (*Fig. 3*).

**Fig. 3:**
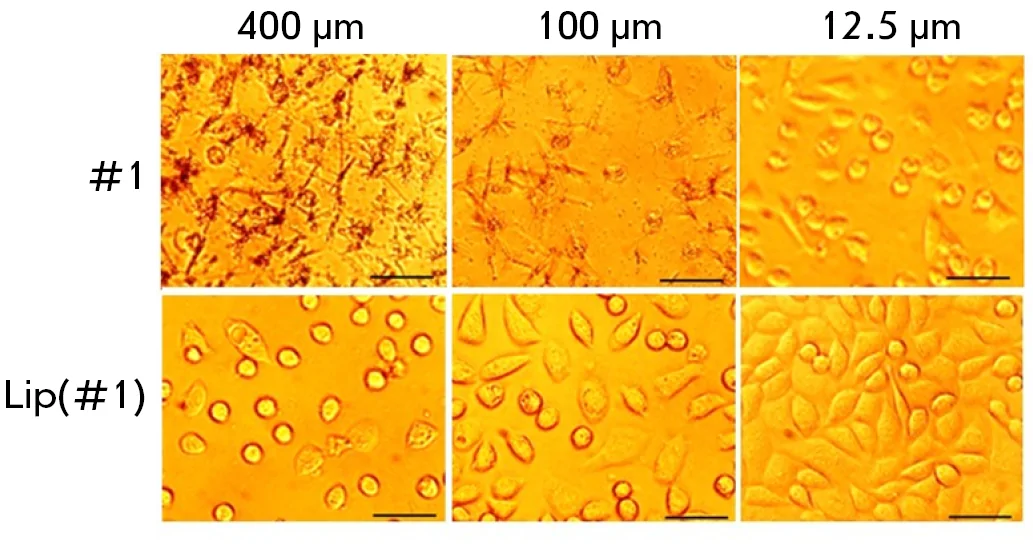
Bright-field microscopy of L929 cells after incubation with free compound #1 (top row) and its liposomal formulation (bottom row). The concentrations of compound #1 are indicated above each row. Scale bar: 50 μm

The presence of DARPin_9-29 molecules highly affine for HER2 on the liposome surface enabled their interaction with the HER2 tumor-associated antigen.

The conjugation of liposomes to DARPin_9-29 slightly increased their hydrodynamic diameter from 116.8 ± 0.6 to 128.3 ± 0.4 nm (*Fig. 4A*), while their ζ-potential remained negative (–5.52 ± 0.48 and –9.03 ± 0.34 mV, respectively), demonstrating that the samples were stable and did not tend to aggregate (*Fig. 4B*).

**Fig. 4:**
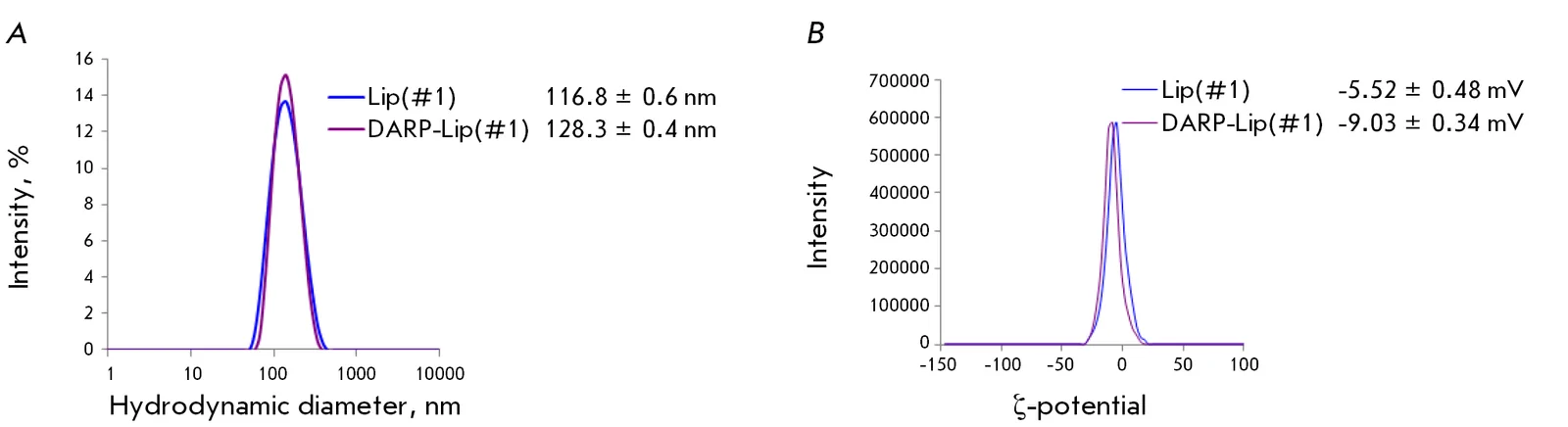
Hydrodynamic diameter (*A*) and ζ-potential (*B*) of liposomes loaded with compound #1: the lilac curve represents the liposomes modified with a targeting module; the blue curve represents unmodified liposomes

**Analysis of the *in vitro* antigenic specificity of DARP-modified liposomes**

The antigenic specificity of the targeted DARPin_9-29 module within the conjugate with liposomes was studied by flow cytometry and confocal microscopy (*Fig. 5*). For visualization, the free DARPin_9-29 and DARP-Lip(#1) were conjugated to the AF-488-NHS fluorescent label according to the procedure described in the Experimental section. Two human carcinoma cell lines were used: the HER2-overexpressing SKOV-3 ovarian cancer cell line (10^6^ receptors per cell) [16, 17] and the HeLa cervical carcinoma cell line characterized by normal HER2 expression (baseline for all epithelial tissues) (10^4^ receptors per cell) [18].

**Fig. 5:**
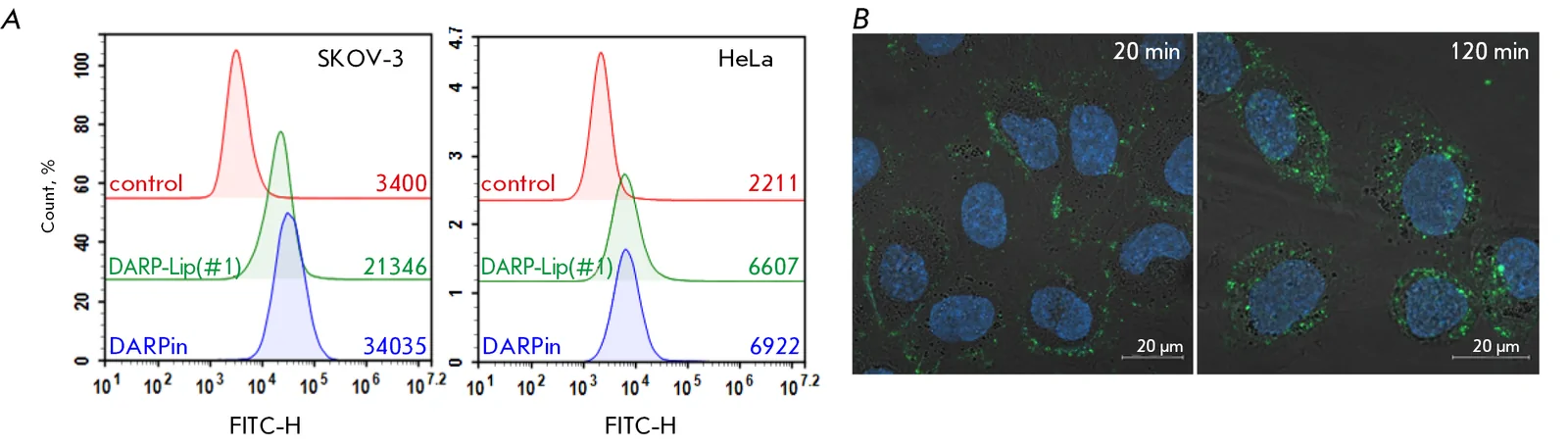
*In vitro* study of the specificity of the interaction between DARP-Lip(#1) and the HER2 receptor using flow cytometry (*A*) and confocal microscopy (*B*). The histograms show the mean fluorescence intensity in the green channel for cells treated with DARPin_9-29 and DARP-Lip(#1) (blue and green curves, respectively) and control cells (red curve). The incubation time of SKOV-3 cells with DARP-Lip(#1) prior to imaging is indicated on the micrographs. Nuclei were stained with Hoechst 33342

The flow cytometry data unambiguously confirm that the *in vitro* conjugation to DARP-Lip(#1) is HER2-specific. Thus, the incubation of HER2-positive SKOV-3 cells with DARPin_9-29 or DARP-Lip(#1) (the blue and green curves in the left histogram in *Fig. 5A*) increased the fluorescence intensity compared to the control (the red curve) ten and sixfold, respectively. The fluorescence intensity of HeLa cells treated with DARPin_9-29 or DARP-Lip(#1) using the same procedure differed from the control only threefold (the right histogram in *Fig. 5A*).

The confocal microscopy data also confirmed the specificity of DARP-Lip(#1) conjugation to the HER2 receptor on the surface of cancer cells. A characteristic green halo appeared on the plasma membrane after short-term (20 min) incubation of SKOV-3 cells with DARP-Lip(#1) (the left micrograph in *Fig. 5B*). Further incubation led to conjugate internalization as proved by the green pixels in the cytoplasm (the right micrograph in *Fig. 5B*).

**Analysis of the *in vitro* cytotoxicity of liposomes with encapsulated compound #1**

The cytotoxicity of the liposomal formulation (targeted and non-targeted) of compound #1 was analyzed by the conventional MTT assay using a HER2-overexpessing SKOV-3cell line and the HeLa cell line with normal HER2 expression.

*Figure 6* shows that the viability of cells treated with DARP-Lip(#1) decreased in a dose-dependent manner; IC_50_ for HER2-positive SKOV-3 cells was 3.7-fold lower than that for HeLa cells characterized by a normal receptor density. The targeting index calculated as the ratio between IC_50_ Lip(#1) and IC_50_ DARP-Lip(#1) was six times higher for the SKOV-3 cell line. The significant difference in this parameter for the cells characterized by low and high HER2 expression levels (3.02 and 18.98, respectively) demonstrates that cytotoxicity was enhanced against the cells with a high target antigen density. It is worth noting that the non-targeted liposomes loaded with compound #1 exhibited a virtually similar cytotoxicity against the studied cell lines, which can be attributed to the identical mechanisms of merging of non-targeted liposomes with the plasma membrane.

**Fig. 6:**
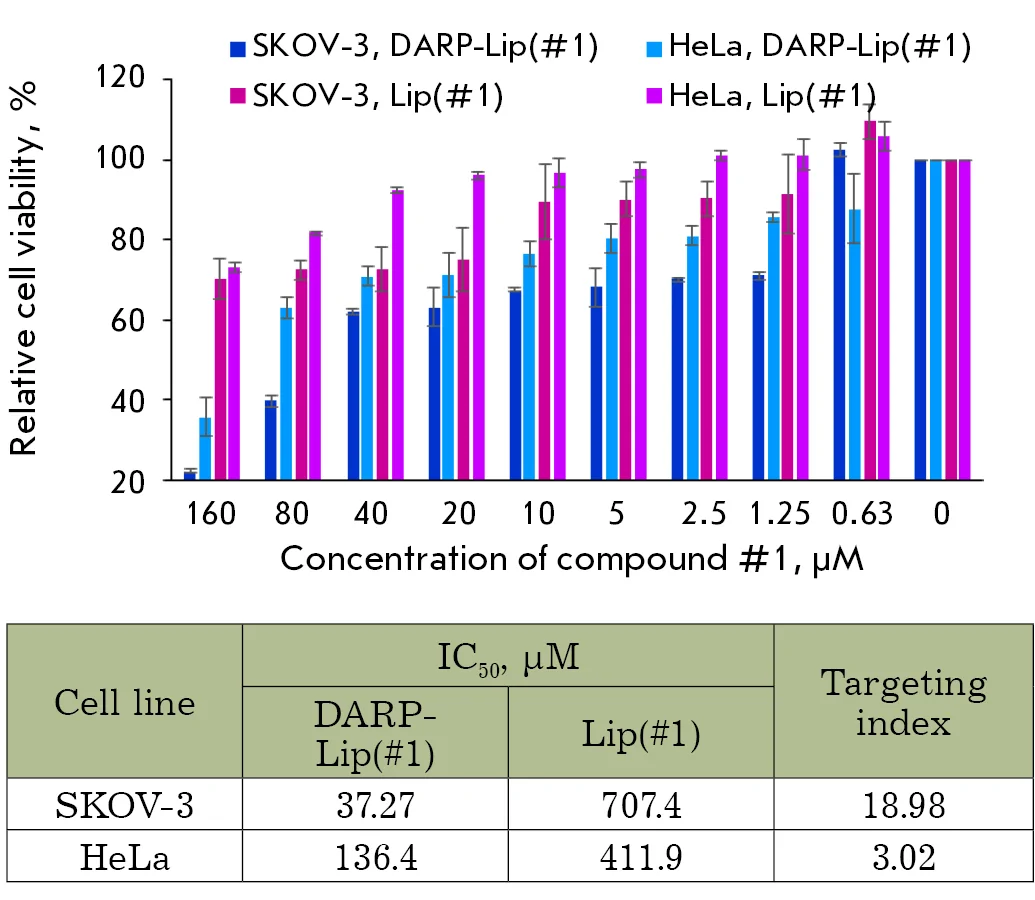
*In vitro* cytotoxicity analysis of DARP-Lip(#1) and Lip(#1) against the SKOV-3 and HeLa cell lines. Error bars represent the standard deviation (SD). IC_50_ values were calculated by data fitting using nonlinear regression with a four-parameter dose–response model (GraphPad Prism 10.1.0)

## CONCLUSION

THIQ derivatives hold great potential as anticancer agents but are characterized by low hydrophilicity, which impedes their *in vivo* applications. Encapsulation of hydrophobic drugs in a lipid matrix prevents their crystallization in aqueous media and enhances bioavailability under physiological conditions.

The targeted liposomes with encapsulated 1-tridecyl-6,7-dimethoxy-1,2,3,4-tetrahydroisoquinoline developed in this study to ensure delivery to HER2-positive cancer cells proved to be stable in aqueous media (*Fig. 3*), capable of effectively interacting with the target antigen *in vitro* (*Fig. 5*), as well as exhibit HER2-specific cytotoxicity. The targeting index for the SKOV-3 cell line characterized by high receptor density was sixfold higher than that for the HeLa cell line with a baseline HER2 expression. The significant difference in the targeting index between the cells characterized by low and high HER2 expression levels (3.02 and 18.98, respectively) demonstrates that the cytotoxicity of the targeted liposomal agent DARP-Lip(#1) is significantly increased against cells with a high target antigen density.

Development of tolerance to anticancer drugs in tumor cells is the major hurdle to successful cancer chemotherapy [19]. This is often related to inhibition of signal transduction by a tumor cell at different levels in the apoptotic self-destruction program via upregulation of the expression of anti-apoptotic genes belonging to the *BCL2* family (*BCL2, BCL-X_L_*, and *MCL-1*), inactivation of the expression of proapoptotic genes (*BAX, APAF1,* and *XAF1*), alterations in the p53 and PI3K/AKT signaling pathways, and overexpression of ATP-binding cassette (ABC) transporters [19, 20, 21].

Isoquinoline derivatives are known to exhibit antiproliferative and pro-apoptotic activities, as they cause cell cycle arrest and induce apoptosis by directly interacting with the proteins involved in apoptosis suppression in cancer cells [22, 23].

Our immediate future research will focus on the efficacy of 1-tridecyl-6,7-dimethoxy-1,2,3,4-tetrahydroisoquinoline against chemo-resistant cancer cells, and our findings may pave the way to developing a highly effective antitumor agent capable of targeting the tumor evasion mechanisms.

## Abbreviations

HER2: human epidermal growth factor receptor type 2
THIQ: 1,2,3,4-tetrahydroisoquinoline
IC50: half-maximal inhibitory concentration (concentration causing 50% cell death)
